# Comparison of preoperative CT- and MRI-based multiparametric radiomics in the prediction of lymph node metastasis in rectal cancer

**DOI:** 10.3389/fonc.2023.1230698

**Published:** 2023-11-24

**Authors:** Yue Niu, Xiaoping Yu, Lu Wen, Feng Bi, Lian Jian, Siye Liu, Yanhui Yang, Yi Zhang, Qiang Lu

**Affiliations:** ^1^ Department of Diagnostic Radiology, Graduate Collaborative Training Base of Hunan Cancer Hospital, Hengyang Medical School, University of South China, Hengyang, Hunan, China; ^2^ Department of Diagnostic Radiology, Hunan Cancer Hospital and The Affiliated Cancer Hospital of Xiangya School of Medicine, Central South University, Changsha, Hunan, China

**Keywords:** rectal cancer, lymph nodes, radiomics, magnetic resonance imaging (MRI), computed tomography (CT)

## Abstract

**Objective:**

To compare computed tomography (CT)- and magnetic resonance imaging (MRI)-based multiparametric radiomics models and validate a multi-modality, multiparametric clinical-radiomics nomogram for individual preoperative prediction of lymph node metastasis (LNM) in rectal cancer (RC) patients.

**Methods:**

234 rectal adenocarcinoma patients from our retrospective study cohort were randomly selected as the training (n = 164) and testing (n = 70) cohorts. The radiomics features of the primary tumor were extracted from the non-contrast enhanced computed tomography (NCE-CT), the enhanced computed tomography (CE-CT), the T2-weighted imaging (T2WI) and the gadolinium contrast-enhanced T1-weighted imaging (CE-TIWI) of each patient. Three kinds of models were constructed based on training cohort, including the Clinical model (based on the clinical features), the radiomics models (based on NCE-CT, CE-CT, T2WI, CE-T1WI, CT, MRI, CT combing with MRI) and the clinical-radiomics models (based on CT or MRI radiomics model combing with clinical data) and Clinical-IMG model (based on CT and MRI radiomics model combing with clinical data). The performances of the 11 models were evaluated via the area under the receiver operator characteristic curve (AUC), accuracy, sensitivity, and specificity in the training and validation cohort. Differences in the AUCs among the 11 models were compared using DeLong’s test. Finally, the optimal model (Clinical-IMG model) was selected to create a radiomics nomogram. The performance of the nomogram to evaluate clinical efficacy was verified by ROC curves and decision curve analysis (DCA).

**Results:**

The MRI radiomics model in the validation cohort significantly outperformed than CT radiomics model (AUC, 0.785 vs. 0.721, *p*<0.05). The Clinical-IMG nomogram had the highest prediction efficiency than all other predictive models (*p*<0.05), of which the AUC was 0.947, the sensitivity was 0.870 and the specificity was 0.884.

**Conclusion:**

MRI radiomics model performed better than both CT radiomics model and Clinical model in predicting LNM of RC. The clinical-radiomics nomogram that combines the radiomics features obtained from both CT and MRI along with preoperative clinical characteristics exhibits the best diagnostic performance.

## Introduction

Rectal cancer (RC) is one of the leading causes of cancer-related deaths. The latest data from GLOBOCAN 2021 reported RC as 8th among all cancers worldwide with high morbidity and mortality (1, 2).There is much evidence that lymph node metastasis (LNM) is the most important and closely correlated with the poor prognosis due to a high rate of local recurrence (3). Thus, preoperative assessment of LNM can provide important information to determine the need for adjuvant therapy and the adequacy of surgical resection (4).

It is well known that both computed tomography (CT) and magnetic resonance imaging (MRI) are common imaging methods to assess Tumor-Node-Metastasis (TNM) staging of RC. MRI has the highest contrast resolution for the soft tissues, allowing the best depiction of relationship between the primary focus of RC and the surrounding anatomical structures, which is very useful in tumor (T) staging (5). It is also valuable in determining nodal (N) staging because of its ability to better show the internal condition of lymph nodes (6). CT can rapidly scan the entire abdomen and chest, allowing for distant metastasis evaluation, as well as T and N staging (2). In fact, traditional imaging methods as CT and MRI both have limited ability to predict LN status with morphological criteria due to the features of metastatic LNs with smaller short diameter are often similar to those of nonmetastatic LNs (7, 8). Additionally, MRI is less accurate in N staging than in T staging with values of sensitivity and specificity ranging between 58–77% and 62–74%, respectively (9). Therefore, improving the technique of preoperative identification of LNM is key imperatives.

Radiomics is a noninvasive method that reveals the heterogeneity of tumors in specific regions of interest (ROI) from medical images (10). Several studies reported radiomics features could predict LNM in other malignant tumors (11, 12). For colorectal cancer, Huang et al. (13) used a contrast enhanced CT (CE-CT)-based radiomics model to discriminate LNM in colorectal cancer with a concordance index (C-index) of 0.736-0.778. Su et al (14) constructed a nomogram model based on T2-weighted imaging (T2WI) radiomics and MRI reported model in RC with an area under the receiver operating characteristic curve (AUC) of 0.891 in the validation group. However, these studies were based on single sequence only, with variable predictive efficacy. As mentioned previously, both CT and MRI are imaging tools for RC staging, but there is lack of evidence that CE-CT or T2WI is the optimal sequence for constructing radiomics model. A comparison of radiomics models between common modalities including non-contrast enhanced CT(NCE-CT), CE-CT, T2WI, gadolinium-enhanced T1-weighted imaging (CE-T1WI) is needed to justify the reasonableness. Therefore, it might be beneficial for the development of a multiparametric radiomics method to assess LNM in RC. In addition, multiparametric data extracted through multi-modality imaging might be complementary to the use of morphological changes (4).

The purpose of this study was to compare CT- and MR-based multiparametric radiomics models and validate a multi-modality, multiparametric clinical-radiomics nomogram based on NCE-CE, CE-CT, CE-T1WI and T2WI that could provide a convenient and rapid tool to accurately predict preoperative LNM in RC.

## Materials and methods

### Patient data

The institutional review board approved the present retrospective study, and the informed consent was waived. A total of 234 patients who underwent radical resection of RC from April 2012 to May 2018 were included in this study, and their preoperative clinical and imaging data were retrospectively analyzed. The inclusion criteria were as follows (1): pathologically confirmed non-mucinous rectal adenocarcinoma after surgery (2), completed baseline MRI and CT examinations before surgery, and (3) patients with LNs with short-axis (SA) diameters≥3 mm on both CT and MRI. The exclusion criteria were as follows (1): received adjuvant therapy such as radiotherapy or chemotherapy before operation (2), incomplete clinical, pathological or imaging data, or (3) obvious motion artifacts caused by breathing or intestinal peristalsis. According to the random distribution of 7:3, patients were divided into two cohorts: a training cohort (n = 164) and a validation cohort (n = 70). The general clinical data including patient age, sex, location of tumor, MRI-reported LNM, MRI-reported extramural vascular invasion (EMVI), carcinoembryonic antigen (CEA), and clinical tumor (cT) stage (the eighth edition of the American Joint Committee on Cancer [AJCC 8th] TNM staging system) were collected and tabulated.

### Image acquisition

All patients were examined by abdominal CT and pelvic (rectum) MRI within 1 week before surgical operation. All MRI examinations were performed with a 3.0-T MRI scanner (Discovery 750W®, GE Healthcare, Waukesha, WI). The MRI sequences included high-resolution T2WI (axial, coronal and sagittal), T1WI (axial), diffusion-weighted imaging (DWI, axial) and CE-T1WI (axial, coronal and sagittal) sequences. For venous phase CE-T1WI, the contrast agent gadodiamide (Omniscan®, GE Medical System, NJ) was intravenously administered at a dose of 0.1 mmol/kg of body weight with a flow rate of 3.5 ml/s using a power injector, followed by a bolus injection of 20 ml of normal saline. NCE-CT and CE-CT images were obtained using a 64-detector (Siemens Somatom Definition AS+) or 256-detector row CT scanner (GE Revolution Xtream). CE-CT scan was performed during the portal venous phase determined with automated scan triggering software and the injection rate of the contrast medium (Omnipaque® 350, GE Medical System, NJ) was 2.5 ml/s. The portal venous-phase scanning automatically began 25 seconds after the trigger attenuation threshold (100 HU) was reached at the level of the descending aorta. Both CE-CT and NCE-CT were acquired covering the same axial extent, during the same acquisition session and in the same scanner for each patient, using the same reconstruction settings (slice thickness and matrix size), reducing any potential misregistration issue between the two datasets. The scanning parameters are provided in the **Supplementary Materials Tables S1** and **S2**.

### Image evaluation

Two experienced radiologists (radiologist A and radiologist B, with experience of 5 and 12 years in the diagnosis of RC, respectively) blinded to the histopathology results reviewed the images in consensus. EMVI positivity on MRI was defined as follows (15) (1): tumor signal intensity in a vascular structure (2), dilated vessels, and (3) tumoral extension through the vessel wall invading the vessel border. Qualitative criteria of MRI reported LNM were based on the 2016 European Society of Gastrointestinal and Abdominal Radiology consensus meeting (7). Disagreements between the two radiologists in the assessment of these features were resolved by the assessment of a third senior radiologist with 20 years of experience in the diagnosis of RC.

### Pathological analysis

All patients underwent total mesorectal excision (TME). We referred to the histopathological assessment of regional LNM as the gold standard. Pathological reports of surgically resected specimensaccording to the AJCC 8th TNM staging system (16). The negative LNM (LNM-) group was defined as patients with no regional LNM. The positive LNM (LNM+) group was defined when patients’ number of regional LNM was greater than or equal to one.

### Tumor segmentation and feature extraction

ROI segmentation was performed on CT and MRI images by ITK-SNAP software (http://www.itksnap.org). Before segmentation, a comprehensive lesion assessment was firstly conducted in the Picture Archiving and Communication System (PACS) by referencing the entire sequence of CT and MRI. For CT, segmentation was based on unenhanced and portal venous phase–enhanced images. While for MRI, segmentation was based on high-resolution T2WI oblique axial sequence and CE-T1WI axial sequence. Since the acquisition of both NCE-CT and CE-CT images was carried out during the same examination, image registration was unnecessary, and it allowed to automatically report the segmentation performed by the radiologist on CE-CT images on the associated NCE-CT images within ITK-SNAP (17). This ensured avoiding differences between features occurring from different segmentations shape and volumes. Segmentations were manually performed on the CE-T1WI and T2WI sequences separately. The above segmentation was completed by radiologist A and radiologist B who were blinded to the pathological information. The ROI was manually sketched along the tumor edge to avoid cystic degeneration, bleeding, and necrosis of the tumor as far as possible. The lesions were double-blindly delineated by two observers in 50 cases randomly selected for different sequences and two sets of segmentation masks were obtained for each sequence. The corresponding radiomics features were calculated according to the segmentation results of the two groups, and intraclass correlation coefficients (ICCs) were calculated to assess the interobserver correlation coefficient reproducibility of the radiomic feature extraction. Next, the segmented image was preprocessed and the N4 correction algorithm in the 3D Slicer (version 4.9, http://www.slicer.org) was used to remove the artifacts of the MRI offset field, reducing the unevenness of the radio-frequency field and the influence of the MR device itself. Then, the gray values of CT and MRI images were normalized to [0-255] to reduce the gray differences between different sequences of different patients, acquisition time and parameter settings so as to ensure the accuracy and reliability of texture analysis. The B-spline interpolation algorithm was used to resample the ROI to a uniform size (1 * 1 * 1 mm^3^) for feature extraction.

The subsequent feature extraction was performed by a radiomic module (backed by Pyradiomics v3.0) embedded in the open-source software package 3D Slicer. Two image filters, wavelet and Laplacian of Gaussian (LoG) were applied to original images, respectively. Sigma parameter of LoG filter defined the texture roughness to be emphasized. We set the sigma size to 1,3 and 5 to obtain filtered images with different textures. In wavelet filter processing, bin width was set to 10. Then, 1130 radiomics features were extracted from the preprocessed images of each mode, including (1) shape-based features (2) histogram features (3) matrix texture features: Grey-level co-occurrence matrix (GLCM), Grey-level run length matrix (GLRLM), Grey-level size zone matrix (GLSZM), Gray-level dependence matrix (GLDM) and Neighbourhood grey-tone difference matrix (NGTDM) (4) wavelet features. These features have been shown to be characteristic of cancer heterogeneity and may reflect changes in image structure (18).

### Feature selection and model building

Feature selection and model construction only used training set data, and validation set was used for model evaluation. Firstly, the features of ICC calculated by segmentation difference were preliminarily screened to eliminate the unstable features with ICC < 0.8. Then, Pearson correlation (PCC) analysis was performed separately for each class of the remaining radiomics features to obtain a feature set with relatively small redundancy (the correlation coefficient threshold was set to 0.99). After the obtained feature set was standardized by Minmax or Zscore algorithm, the recursive feature elimination (RFE) algorithm was used to find the optimal feature subset. Five classification algorithms (Random forest, Gaussian process, Adaboost, K-Nearest Neighbor and Multi-layer Perceptron) were compared to obtain the optimal one. The 5-fold cross validation was used for feature selection and classification algorithm optimization, and the optimal radiomics model was constructed and evaluated in the independent validation set.

To quantify the accuracy of the signature constructed by different radiomics models, we calculated the LN-positive probability score of each case using the radiomics formula of the training set, which was defined as the radiomics score (rad-score). Then, univariate logistic regression was used to identify potential predictors of clinical risk factors for LNM. For the multivariable analysis, first, all the significant clinical characteristics were included, and next, only the variables with P <0.10 at univariable analysis were included and selected using the backward elimination process. The Clinical model and clinical-radiomics nomogram for predicting LNM were constructed using the selected clinical predictors.

The Hosmer–Lemeshow test was performed to assess the goodness-of-fit of the nomogram. Calibration curves were generated to evaluate the calibration of the nomogram. The AUC values were calculated to assess the discrimination performances of the Clinical model, radiomics models and clinical-radiomic nomogram for predicting LNM. The clinical utility was evaluated by decision curve analysis (DCA).

### Statistical analysis

SPSS 26.0 (IBM) and R software (version 2.15.3) were used for statistical analysis. The baseline characteristics of patients with RC were compared using Student’s t test, nonparametric test, chi-squared test, and Fisher’s exact test (where appropriate). The diagnostic performance was compared by receiver operating characteristic (ROC) analysis, and the difference in AUC values between these models was compared using Delong’s test.

## Results

### Patient characteristics

**Figure 1:**
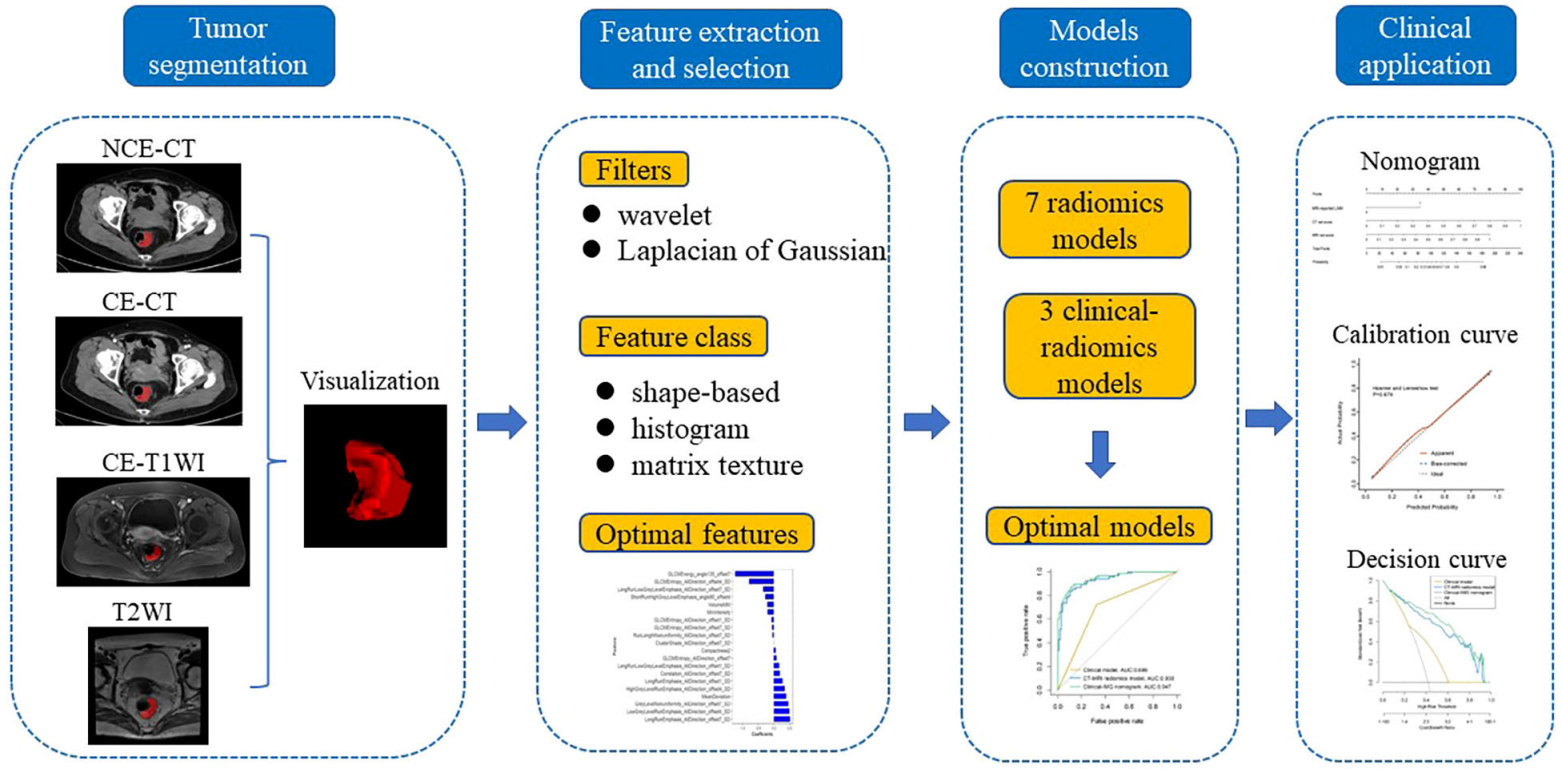
Research flow chart of the radiomics models.

The research flow chart is shown in **Figure 1**. There are no significant differences in any of clinical features between the training and validation cohorts, as shown in **Table 1**. There are significant differences in MRI-reported LNM and EMVI between the LNM- and LNM+ groups both in the training and validation cohorts. Significant difference in cT stage was found only in the validation cohorts. See **Table 2** for details.

**Table 1:** Baseline characteristics of the study population.

Characteristic	Level	Training Cohort (n=164)	Validation Cohort (n=70)	*p* value
Age (years)		60.8 ± 9.7	60.8 ± 10.0	0.982^a^
Sex (No.)				0.584^b^
	Male	100(61.0)	40(57.1)	
	Female	64(39.0)	30(42.9)	
Location				0.096^b^
	lower	68(41.5)	19(27.1)	
	middle	71(43.3)	37(52.9)	
	upper	25(15.2)	13(18.6)	
	≥2 parts	0(0.0)	1(1.4)	
cT stage				0.098^b^
	T_1-2_	42(25.6)	11(15.7)	
	T3-4	122(74.4)	59(84.3)	
MRI-reported LNM				0.753^b^
	Negative	83(50.6)	37(52.9)	
	Positive	81(49.4)	33(47.1)	
MRI-reported EMVI				0.677^b^
	Negative	106(64.6)	48(68.6)	
	Positive	19(11.6)	9(12.9)	
	NA	39(23.8)	13(18.6)	
CEA (mg/l)		2.3(1.4,4.6)	2.2(1.5,6.6)	0.402^c^

a Variables were tested using independent sample t-test, data are mean ± SD.

b Variables were tested using Chi-square test or Fisher’s exact test, data are the number of patients, with percentages in parentheses.

c Variables were tested using Mann–Whitney U-test, data are median, with Interquartile range in parentheses.

SD, standard deviation; LNM, lymph node metastasis; PLNM, pathological-reported lymph node metastasis; EMVI, extramural vascular invasion; CEA, carcinoembryonic antigen.

**Table 2:** Clinical characteristics of the training and validation cohorts.

Characteristic	Level	Training Cohort (n=164)			Validation Cohort (n=70)		
		PLNM (–)	PLNM(+)	*p* value	PLNM (–)	PLNM(+)	*p* value
		(n=95)	(n=69)		(n=41)	(n=29)	
Age (years)		61.3 ± 9.6	60.2 ± 9.8	0.482^a^	61.0 ± 10.1	60.5 ± 9.7	0.833^a^
Sex (No.)				0.501^b^			0.779^b^
	Male	60(63.2)	40(58.0)		24(58.5)	16(55.2)	
	Female	35(36.8)	29(42.0)		17(41.5)	13(44.8)	
Location				0.392^b^			0.635^b^
	lower	43(45.3)	25(36.2)		12(29.3)	7(24.1)	
	middle	40(42.1)	31(44.9)		22(53.7)	15(51.7)	
	upper	12(12.6)	13(18.8)		7(17.1)	6(20.7)	
	≥2 parts	0(0.0)	0(0.0)		0(0.0)	1(3.5)	
cT stage				0.333^b^			0.018^b^*
	T_1-2_	27(28.4)	15(21.7)		10(24.4)	1(3.4)	
	T3-4	68(71.6)	54(78.3)		31(75.6)	28(96.6)	
MRI-reported LNM				<0.001^b^*			0.010^b^*
	Negative	64(67.4)	19(27.5)		27(65.9)	10(34.5)	
	Positive	31(32.6)	50(72.5)		14(34.1)	19(65.5)	
MRI-reported EMVI				0.043^b^*			0.022^b^*
	Negative	64(67.4)	42(60.9)		29(70.7)	19(65.5)	
	Positive	6(6.3)	13(18.8)		8(19.5)	1(3.5)	
	NA	25(26.3)	14(20.3)		4(9.8)	9(31.0)	
CEA (mg/l)		2.1(1.3,3.3)	2.5(1.5,6.7)	0.077^c^	2.0(1.4,4.7)	3.2(1.7,9.5)	0.076^c^

a Variables were tested using independent sample t-test, data are mean ± SD.

b Variables were tested using Chi-square test or Fisher’s exact test, data are the number of patients, with percentages in parentheses.

c Variables were tested using Mann–Whitney U-test, data are median, with Interquartile range in parentheses.

*p values less than 0.05 were considered statistically significant.

SD, standard deviation; LNM, lymph node metastasis; PLNM, pathological-reported lymph node metastasis; EMVI, extramural vascular invasion; CEA, carcinoembryonic antigen.

NA, Not Applicable.

### Diagnostic performance of MRI-reported LNM

Diagnostic performance of MRI-reported LNM is summarized in **Supplementary Materials Table S3**. The MRI-reported LNM achieved a sensitivity of 70.4%, a specificity of 66.9%, an accuracy of 68.4%, a positive predictive value of 60.5%, a negative predictive value of 75.8%, and an AUC of 0.687 (95% confidence interval 0.617-0.756).

### Rad-score evaluation

As is shown in **Supplementary materials**, the remaining 12, 3,11and 6 features after dimensionality reduction were extracted from NCE-CT, CE-CT, CE-T1WI and T2WI respectively. The diagnostic performance of the radiomics signature in the training cohort and validation cohort are as shown in **Tables 3A, B**, respectively. We combined NCE-CT radiomics model with CE-CT radiomics model to obtain CT radiomics model, and combined CE-T1 radiomics model with T2 radiomics model to obtain MRI radiomics model. Then, we combined CT radiomics model with MRI radiomics model to obtain CT-MRI radiomics model. In terms of predictive efficacy, the CT-MRI radiomics model significantly outperforms the remaining six radiomics models, with AUCs of 0.930 and 0.802 for the training and validation sets, respectively. The comparison of predictive efficacy between different radiomics models is shown in **Table 4**.

**Table 3:** Diagnostic efficacy of different models. .

A. Training cohort						
	AUC (95%CI)	*p*-value	Sensitivity	Specificity	Accuracy	
Clinical model	0.699(0.667-0.731)	Ref.	0.725	0.674	0.695	
NCE-CT_rad signature	0.646(0.609-0.683)	0.033*	0.565	0.589	0.579	
CE-CT_rad signature	0.886(0.865-0.908)	< 0.001*	0.754	0.789	0.774	
CT_rad signature	0.888(0.866-0.909)	< 0.001*	0.841	0.758	0.793	
CE-T1_rad signature	0.726(0.690-0.762)	0.256	0.609	0.779	0.707	
T2_rad signature	0.792(0.764-0.819)	< 0.001*	0.667	0.789	0.738	
MRI_rad signature	0.848(0.821-0.874)	< 0.001*	0.797	0.747	0.768	
CT-MRI_rad signature	0.930(0.913-0.947)	< 0.001*	0.841	0.895	0.872	
Clinical-CT nomogram	0.920(0.903-0.937)	< 0.001*	0.899	0.789	0.835	
Clinical-MRI nomogram	0.879(0.855-0.903)	< 0.001*	0.768	0.789	0.780	
Clinical-IMG nomogram	0.947(0.933-0.962)	< 0.001*	0.870	0.884	0.878	
B. Validation cohort						
	AUC (95%CI)	*p*-value	Sensitivity	Specificity	Accuracy	
Clinical model	0.657(0.624-0.689)	Ref.	0.655	0.659	0.657	
NCE-CT_rad signature	0.676(0.640-0.712)	0.410	0.690	0.537	0.600	
CE-CT_rad signature	0.711(0.676-0.746)	0.019*	0.690	0.610	0.643	
CT_rad signature	0.721(0.687-0.756)	0.005*	0.759	0.610	0.671	
CE-T1_rad signature	0.735(0.701-0.769)	< 0.001*	0.586	0.659	0.629	
T2_rad signature	0.728(0.698-0.758)	< 0.001*	0.414	0.854	0.671	
MRI_rad signature	0.785(0.754-0.815)	< 0.001*	0.621	0.732	0.686	
CT-MRI_rad signature	0.802(0.772-0.831)	< 0.001*	0.621	0.78	0.714	
Clinical-CT nomogram	0.744(0.710-0.777)	< 0.001*	0.690	0.659	0.671	
Clinical-MRI nomogram	0.796(0.766-0.827)	< 0.001*	0.690	0.780	0.743	
Clinical-IMG nomogram	0.828(0.799-0.856)	< 0.001*	0.759	0.780	0.771	

CI, confidence intervals; AUC, the area under the receiver operating characteristic curve.

*p values less than 0.05 were considered statistically significant.

**Table 4:** Comparison of prediction performance among different models in validation cohort.

	Clinical model	NCE-CT_rad signature	CE-CT_rad signature	CT_rad signature	CE-T1_rad signature	T2_rad signature	MRI_rad signature	CT-MRI_rad signature	Clinical-CT nomogram	Clinical-MRI nomogram	Clinical-IMG nomogram
Clinical model	–										
NCE-CT_rad signature	0.410	–									
CE-CT_rad signature	0.019*	0.187	–								
CT_rad signature	0.005*	<0.001*	0.081	–							
CE-T1_rad signature	< 0.001*	0.011	0.345	0.581	–						
T2_rad signature	0.002*	0.039*	0.476	0.765	0.780	–					
MRI_rad signature	< 0.001*	< 0.001*	0.002*	0.007*	< 0.001*	< 0.001*	–				
CT-MRI_rad signature	< 0.001*	< 0.001*	< 0.001*	< 0.001*	0.001*	< 0.001*	0.318	–			
Clinical-CT nomogram	< 0.001*							< 0.001*	–		
Clinical-MRI nomogram	< 0.001*							0.768	0.009*	–	
Clinical-IMG nomogram	< 0.001*	< 0.001*	< 0.001*	< 0.001*	< 0.001*	< 0.001*	0.011*	< 0.001*	< 0.001*	0.029*	–

*p values less than 0.05 were considered statistically significant.

### Development and evaluation of the clinical-radiomic nomogram

Logistic regression analysis showed that only the MRI-reported LNM was the independent predictors of LNM, as shown in **Table 5**. The Clinical model was constructed using MRI-reported LNM. The clinical-radiomic combined models were constructed by adding CT or MRI radiomics model to the Clinical model. We constructed three clinical-radiomic nomograms, as shown in **Table 3**, including the Clinical-CT nomogram obtained by fusing the Clinical model with CT radiomics model, the Clinical-MRI nomogram obtained by fusing the Clinical model with MRI radiomics model, and the Clinical-IMG nomogram constructed by fusing the Clinical model with the CT and MRI radiomics models. In terms of the prediction effect, the Clinical-IMG nomogram is significantly higher than the other two nomograms, with AUCs of 0.947 and 0.828 for the training and validation sets, respectively. The comparison of predictive efficacy between different nomograms is shown in **Table 4**.

**Table 5:** Stepwise logistic regression analysis of LNM prediction.

Variable	Univariate logistic regression	
	OR (95%CI)	*p* value
Sex	1.234(0.603-2.525)	0.565
Age	0.982(0.946-1.019)	0.332
Location	1.907(0.834-4.364)	0.126
Distance from the anus	0.937(0.767-1.144)	0.522
cT stage	0.958(0.422-2.172)	0.918
MRI-reported LNM	5.433(2.751-10.728)	0.000*
MRI-reported EMVI	0.913(0.602-1.383)	0.667
CEA (mg/l)	1.017(0.973-1.062)	0.459

*p values less than 0.05 were considered statistically significant.

LNM, lymph node metastasis; EMVI, extramural vascular invasion; CEA, carcinoembryonic antigen.

### Comparison of prediction performance between different models

The comparison of the predictive efficacy among different models in the validation cohort is shown in **Table 4**. We found that except for NCE-CT radiomics model, the radiomics models were overall superior to the Clinical model (*p*<0.05); the combined multi-parameter models were overall superior to the single-parameter model for both CT and MRI. The CT radiomics model was significantly superior to the NCE-CT radiomics model (0.721 vs 0.676, *p*<0.001) and was slightly outperformed the CE-CT radiomics model (0.721 vs 0.711, *p*=0.081), though there was no statistical significance by Delong test. The MRI radiomics model significantly outperformed the CE-T1WI radiomics model (0.785 vs 0.735, *p*<0.001) or T2WI radiomics model (0.785 vs 0.728, *p*<0.001). The MRI radiomics model significantly outperformed CT radiomics model (0.785 vs 0.721, *p*=0.007). The multimodality multiparametric radiomics models combined with Clinical model to build the Clinical-IMG nomogram had the highest predictive value and significantly outperformed all other radiomics models and nomograms, *p*<0.05.

**Figure 2:**
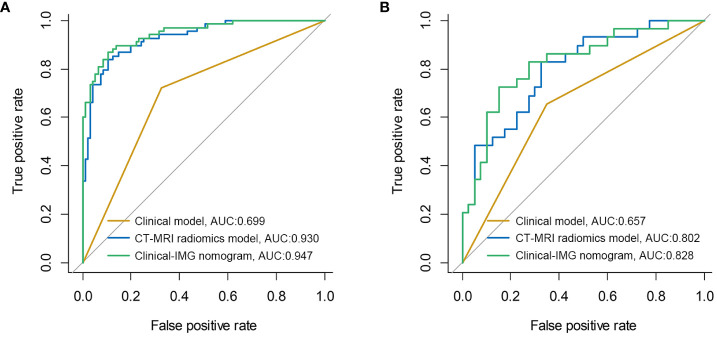
ROC curves of three models in the training cohort **(A)** and validation cohort **(B)**. The results show that the Clinical-IMG nomogram has the highest AUC value.

**Figure 2** shows the ROC curves and AUC of the Clinical model, the optimal radiomics model CT-MRI radiomics model and the optimal clinical-radiomic nomogram Clinical-IMG nomogram in the training (**Figure 2A**) and validation sets (**Figure 2B**). The CT-MRI radiomics model is significantly superior to the Clinical model in both the training and validation sets (AUC,0.930 vs. 0.699, *p*<0.001,0.802 vs. 0.657, *p*<0.001), and the Clinical-IMG nomogram is significantly better than the Clinical model in both the training and validation sets (AUC,0.947 vs. 0.699, *p*<0.001, 0.828vs0.657, *p*<0.001) or CT-MRI radiomics model (AUC,0.947 vs. 0.930, *p*<0.001, 0.828 vs. 0.802, *p*<0.001).

**Figure 3:**
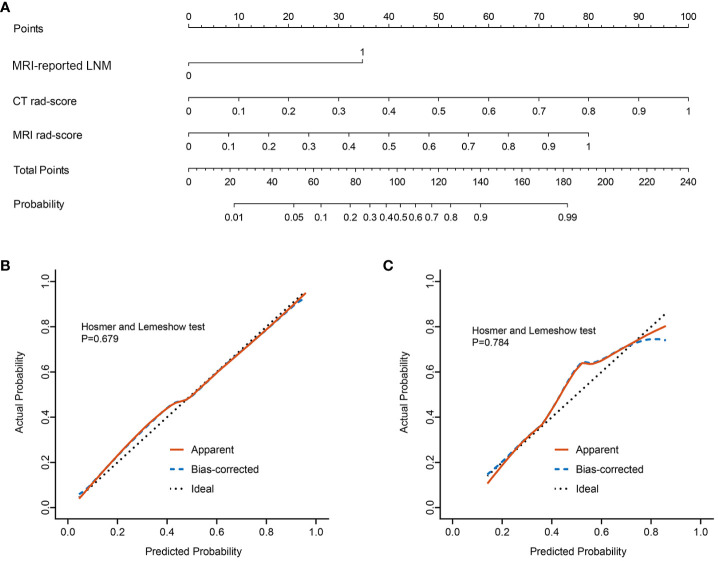
Clinical-IMG nomogram for predicting LNM **(A)**. In the nomogram, a vertical line was drawn according to the value of the rad-score to determine the corresponding value of points. The points of MRI-reported LNM can also be determined in the same way. The total points were the sum of the three points above. Finally, a vertical line was drawn according to the value of the total points to determine the probability of LNM. The calibration curve of the Clinical-IMG nomogram for LNM in the training cohort **(B)** and validation cohort **(C)**. The x-axis represented the predicted LNM risk. The y-axis represented the actual LNM rate. A diagonal dotted line indicated the reference line where an ideal nomogram would lie. A red solid line indicated the performance of the nomogram, while the blue dashed line indicated bias correction in the nomogram.

**Figure 4:**
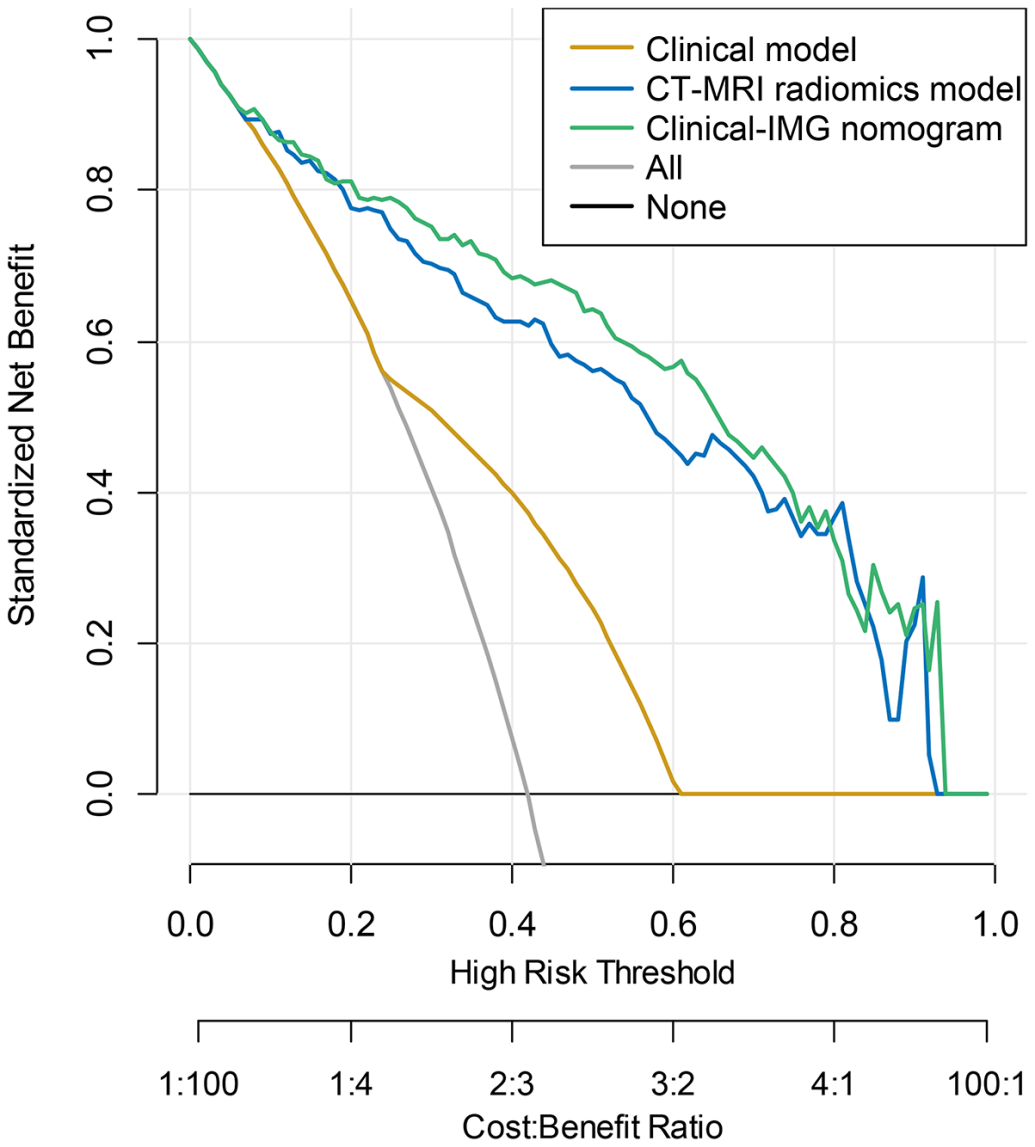
DCA curve for the Clinical-IMG nomogram, CT-MRI radiomics model and Clinical model predicting LNM in the validation cohort. The y-axis indicated the net benefit. The grey line and horizontal black line represented the net benefit of treat-all strategy and treat-none strategy, respectively. The graphs showed that the Clinical-IMG nomogram had the greatest net benefit.

The nomogram was constructed for visualizing the Clinical-IMG combined model, as shown in **Figure 3A**. The calibration curves of the Clinical-IMG nomogram in the training and validation cohort are shown in **Figures 3B, C**, and **4** shows the DCA results. DCA indicates that the Clinical-IMG nomogram has the best clinical net benefit compared with the Clinical model or CT-MRI radiomics model and good calibration in training cohort and validation cohort is identified using the Hosmer–Lemeshow test (all *p*>0.05).

## Discussion

Preoperative noninvasive assessment of LNM in RC patients has always been a hot and difficult issue (19, 20). In the current study, we compared various clinical and radiomics models based on preoperative data of RC patients to predict LNM. The results showed that the radiomics models were generally better than the Clinical model. Also, we constructed several radiomics prediction models from CT or MRI with different parameters. The results indicated that MRI radiomics model was superior to CT radiomics model and the combined multiparametric radiomics model showed better prediction efficiency than the single parameter radiomics models for both CT and MRI. Moreover, we found that the multimodality multiparametric radiomics signatures combined with clinical data build the radiomics nomogram which had the best predictive performance. Together, either CT- or MR-based radiomics techniques can help to non-invasively predict LNM of RC, and the predictive efficacy can be further enhanced when combined with clinical features.

In the present study, radiomics signature of NCE-CT did not have a statistically significant difference from that of the CE-CT in the validation cohort (AUC, 0.676 vs 0.711, *p*=0.187). Yuan et al. (21) recruited 788 patients with RC to construct a CT-based radiomics analysis to predict LNM in RC. It was found that there were no statistical significances of the intra-tumoral Bayes model among the non-enhanced, arterial or venous–phase CT in the training and validation cohorts, which was consistent with our findings. The above findings suggested that NCE-CT and CE-CT radiomics models were of comparable value in predicting LNM of RC. Badic et al. (17) found that some second and third-order textural features extracted from images of patients with primary colorectal tumors were found highly correlated between CE-CT and NCE-CT, and some radiomics features with moderate correlation had potential complementary value for predicting survival outcomes, which may explain the reason of our result that no significant statistical difference between NCE-CT and CE-CT radiomics models may be due to a certain degree of correlation between NCE-CT and CE-CT radiomics features. Considering the safety and economy, avoiding the adverse reactions caused by contrast medium and thus applicable to a wider population (22), the NCE-CT-based radiomics signature is more suitable for predicting LNM in RC than CE-CT. In our data, we also found that the CT radiomics model outperformed the NCE-CT radiomics model (AUC, 0.721 vs 0.676, *p*<0.001), indicating that CT radiomics model combined NCE-CT with CE-CT can further improve the prediction efficiency. Hence, if appropriate, a CT radiomics model combining multiple parameters would be selected for LNM prediction in RC when possible.

In the current study, there was no significant difference in the predictive value of the MRI radiomics models between CE-T1WI and T2WI in the validation cohort (0.735 vs 0.728, *p* = 0.780). To the best of our knowledge, up to now, it seems none of the studies have compared the efficacy of CE-T1WI and T2WI-based radiomics models in predicting LNM of RC. However, some studies (23, 24) on predicting other high-risk prognostic factors of RC showed similar findings with our work. Zhang et al. (25) and Liu et al. (26) found no significant difference between the radiomics signatures based on CE-T1WI and T2WI in predicting perineural invasion and EMVI of RC, respectively. In clinical practice, acquiring T2WI images instead of CE-T1WI images can avoid adverse effects caused by contrast agents to reduces medical risks and medical expenses. Additionally, it is generally believed that T2WI is preferred for the morphological evaluation of LN while CE-T1WI provides minimal benefit for the accurate determination of metastatic nodes in RC (27). Thus, our study provides a further rationale for the fact that T2WI is often used as an optimal sequence in constructing radiomics model to predict LNM in RC (28–30). Our study also found that the MRI radiomics model significantly outperformed the CE-T1WI radiomics model (0.785 vs. 0.735, *p*<0.001) and T2WI radiomics model (0.785 vs. 0.728, *p*<0.001), which was consistent with the findings obtained in radiomics prediction models for LNM in other types of cancer (31, 32). Deng et al. (33) developed a radiomics predictive model for LNM in cervical cancer using radiomic features extracted from CE-T1WI and T2WI images, which exhibited high performance in LNM prediction. However, they did not compare the differences in predictive efficacy among different MRI parameter sequences. Our research further confirmed that the model constructed from the combination of multiple MRI sequences was superior to single-sequence models in discriminating LN status.

Both CT or MRI could be used for constructing radiomics models and have obtained good predictive efficacy in RC LN status (20). Our study found that the AUC of MRI radiomics model in the validation cohort was significantly higher than that of CT (0.785 vs. 0.721, *p*<0.05), reflecting that MRI-based radiomics is more suitable than CT for assessing N staging, which may be due to the high soft tissue resolution and multiparametric features of MRI that provide more tissue information and allow for a more comprehensive description of the tumor whereas CT reflects only the density differences between tissues (34–36). Additionally, we also found no statistical difference between MRI radiomics model and CT-MRI radiomics model in the validation cohort (0.785 vs. 0.802, *p*=0.318), which indicated that CT radiomics model provided limited value in CT-MRI radiomics model.

Previous researches have demonstrated an association between MRI-reported LNM and the pathological status of LNs (37). The diagnostic performance of MRI-reported LNM in our study was consistent with previous studies (9, 38). In the present study, MRI-reported LNM was incorporated as the only independent predictor for LNM in Clinical model. Except for NCE-CT, the predictive efficacy of different radiomics models were better than that of Clinical model in our study (*p*<0.05), which is consistent with the MRI findings of Li et al (39). These observations indicated that imaging approaches can provide more information relevant to LNM and are always superior to clinical method in evaluating the status of lymphatic metastasis in RC.

Due to the significance of LNM as a crucial prognostic factor impacting local recurrence and overall survival in patients with RC, the preoperative prediction of LNM is highly imperative (29), especially for patients who cannot undergo surgery or biopsy to obtain pathological results because of various causes. In our study, the Clinical, CT and MRI radiomics models were conducive to predict LNM of RC. In order to fully exploit medical information and facilitate clinical applications, we constructed an integration nomogram (Clinical-IMG nomogram) combining the CT and MRI radiomics features with clinical characteristics for the preoperative assessment of LNM in RC, and achieved a better prediction performance. We found that Clinical-IMG nomogram had the highest AUC and better predictive efficacy than all other predictive models (*p*<0.05). Similar to our observation, Li et al. corroborated that clinical-radiomic nomograms outperform clinical models in predicting preoperative LNM (40). Therefore, the development of Clinical-IMG nomogram can enhance the clinical assessment performance of MRI on detecting LNM. In clinical practice, we recommend to establish integration model that incorporates as much multimodality/multiparametric image data and clinical information as possible to achieve continue improvement in the non-invasive prediction of LNM in patients with RC. Additionally, the Clinical-IMG nomogram, as a scoring system, can quantify the probability of LNM to realize the individualized preoperative prediction of LNM risk in RC by clinicians, which is in line with the current development trend of individualized precision medicine (41).

When considering the methods of the present study, there were some limitations. First, this study was retrospectively. However, eligible patients were consecutively retrieved from a prospective database that included all patients with RC in our hospital. Second, our data are limited to a single center study, so our results may not be extended to other medical centers. In the future, multicenter studies are needed to further verify the results of this study. Third, even though diffusion-weighted imaging (DWI) is routinely included in rectal MRI protocols and offers several benefits in various applications, it also has multiple possible shortcomings. Manual drawing of ROIs onto the tumor for quantitative or qualitative assessment may result in interobserver variation. Furthermore, image distortion due to artifacts is common on DWI, particularly around air tissue interfaces. These shortcomings may interfere with radiologists in drawing tumor ROI. Forth, our study has limitations concerning the generalizability of radiomics. Some radiomics features are difficult to explain and have unequivocal significance in clinical practice. Currently, radiomic prediction models are challenging to reproduce and generalize in clinical practice due to their complex processes. Fifth, radiomics features were extracted from the primary tumor rather than LNs in our study. The segmentation of LNs poses challenges due to their relatively small size, potentially unclear boundaries and complex surrounding structures. However, direct identification of LNM remains a direction for the further in-depth research.

In conclusion, MRI radiomics model performs better than both CT radiomics model and Clinical model in predicting LNM of RC. The clinical-radiomics nomogram that combines the radiomics features obtained from both CT and MRI along with preoperative clinical characteristics exhibits the best diagnostic performance. In practice, integrating all possible clinical and imaging information helps to achieve the best model prediction performance.

## Data availability statement

The raw data supporting the conclusions of this article will be made available by the authors, without undue reservation.

## Ethics statement

The studies involving humans were approved by Medical Ethics Committee of Hunan Cancer Hospital. The studies were conducted in accordance with the local legislation and institutional requirements. The ethics committee/institutional review board waived the requirement of written informed consent for participation from the participants or the participants’ legal guardians/next of kin because this retrospective study was approved by the ethics committee of our institution, and informed consent was not required.

## Author contributions

All authors listed have made a substantial, direct, and intellectual contribution to the work and approved it for publication.
