# Supplementary material for: Comparison of preoperative CT- and MRI-based multiparametric radiomics in the prediction of lymph node metastasis in rectal cancer
Source: Front Oncol. 2023 Nov 24;13:1230698. doi: 10.3389/fonc.2023.1230698 (PMC10708912; doi:10.3389/fonc.2023.1230698)
Supplement: Supplementary file 1 [file DataSheet_1.docx]

Supplementary Material

**Comparison of preoperative CT- and MRI-based multiparametric radiomics in the prediction of lymph node metastasis in rectal cancer**

Yue Niu^1,2^, Xiaoping Yu^1,2*^, Lu Wen^2^, Feng Bi^2^, Lian Jian^2^, Siye Liu^2^, Yanhui Yang^1,2^, Yi Zhang^1,2^, Qiang Lu^2^

1 Department of Diagnostic Radiology, Graduate Collaborative Training Base of Hunan Cancer Hospital, Hengyang Medical School, University of South China, Hengyang, Hunan, 421001, China

2 Department of Diagnostic Radiology, Hunan Cancer Hospital, Changsha 410013, Hunan, P.R. China

**Correspondence Author Info:**

Name: Yu Xiaoping

Street address: 283 Tongzipo Road, Yuelu District, Changsha, 410013, Hunan, China

E-mail address: yuxiaoping@hnca.org.cn.

Telephone number: +86 13607313419

Fax number: +86 731 89762577

**I.** **MRI and CT scan parameters**

All MRI examinations were performed with a 3.0-T MRI scanner (Discovery 750W®, GE Healthcare, Waukesha, WI), and CT scans were obtained using a 256-detector row MDCT scanner (Revolution Xtream®, GE Healthcare, Waukesha, WI). The specific scanning parameters are shown in Table S1 and Table S2.

**Table S1. MRI scan parameter list**

| Sequence name | TR(ms) | TE(ms) | slice thickness(mm) | slice space(mm) | FOV(mm) | acquisition matrix | number of excitations |
| --- | --- | --- | --- | --- | --- | --- | --- |
| T1WI(axial) | 4694 | 102 | 5 | 1 | 380 | 320 × 224 | 2 |
| HR-T2WI (oblique axial) | 4121 | 126 | 3 | 1 | 180 | 320 × 320 | 2 |
| HR-T2WI (sagittal) | 2600 | 126 | 4 | 1 | 240 | 416 × 384 | 2 |
| HR-T2WI (oblique coronal) | 2600 | 126 | 3 | 1 | 180 | 320 × 320 | 2 |
| DWI(axial) | 4243 | 73.4 | 4 | 1 | 320 | 128 × 130 | 2 |
| CE-T1WI (axial) | 4.6 | 1.1 | 3 | 0 | 380 | 260 × 224 | 1 |
| CE-T1WI (sagittal) | 4.4 | 2.1 | 3 | 0 | 320 | 260 × 200 | 1 |
| CE-T1WI (coronal) | 4.6 | 1.1 | 4 | 0 | 380 | 260 × 224 | 1 |

**Table S2. CT scan parameter list**

| Scan mode | Tube voltage(kVp) | Tube current (mA) | Gantry rotation speed (s/r) | Pitch | Width of detector (mm) | FOV(mm) | Slice thickness (mm) | Slice gap (mm) |
| --- | --- | --- | --- | --- | --- | --- | --- | --- |
| NCE-CT | 120 | 150-550 | 0.5 | 0.992 | 160 | 360 | 5 | 5 |
| CE-CT | 120 | 150-550 | 0.5 | 0.992 | 160 | 360 | 5 | 5 |

**Ⅱ. Information of Dimension reduction**

1. **NCE-CT** **radiomics signature**

The features of intraclass correlation coefficient (ICC) calculated by segmentation difference were initially screened to obtain 839 features with ICC ≥ 0.8. Then, Pearson correlation (PCC) analysis was performed on the remaining radiomics features to obtain a feature set with relatively small redundancy (the correlation coefficient threshold was set to 0.99) and 477 features were obtained. After standardizing the obtained feature set by Minmax or Zscore algorithm, the recursive feature elimination (RFE) algorithm was used to find the optimal feature subset. The final model contained 12 features and the classifier was Gaussian process (GP).

| log-sigma-3-0-mm-3D_ngtdm_Busyness |
| --- |
| log-sigma-3-0-mm-3D_ngtdm_Strength |
| wavelet-LHH_firstorder_Energy |
| wavelet-LHH_firstorder_Kurtosis |
| wavelet-HLL_glszm_LargeAreaEmphasis |
| wavelet-HLL_glszm_ZoneVariance |
| wavelet-HLH_firstorder_Energy |
| wavelet-HLH_firstorder_Kurtosis |
| wavelet-HLH_glszm_ZoneVariance |
| wavelet-HHL_glszm_LargeAreaEmphasis |
| wavelet-HHH_firstorder_Energy |
| wavelet-LLL_firstorder_Kurtosis |

**2．CE-CT radiomics signature**

The features of ICC calculated by segmentation difference were initially screened to obtain 969 features with ICC ≥ 0.8. Then, PCC analysis was performed on the remaining radiomics features to obtain a feature set with relatively small redundancy (the correlation coefficient threshold was set to 0.99) and 568 features were obtained. After standardizing the obtained feature set by Minmax or Zscore algorithm, the RFE algorithm was used to find the optimal feature subset. The final model contained 3 features and the classifier was Adaboost.

| log-sigma-5-0-mm-3D_glszm_LargeAreaEmphasis |
| --- |
| log-sigma-5-0-mm-3D_glszm_ZoneVariance |
| wavelet-LLL_glszm_ZoneVariance |

1. **CE-T1WI radiomics signature**

The features of ICC calculated by segmentation difference were initially screened to obtain 1036 features with ICC ≥ 0.8. Then, PCC analysis was performed on the remaining radiomics features to obtain a feature set with relatively small redundancy (the correlation coefficient threshold was set to 0.99) and 601 features were obtained. After standardizing the obtained feature set by Minmax or Zscore algorithm, the RFE algorithm was used to find the optimal feature subset. The final model contained 11 features and the classifier was Multi-layer Perceptron (MLP).

| log-sigma-1-0-mm-3D_glszm_SmallAreaEmphasis |
| --- |
| log-sigma-5-0-mm-3D_firstorder_InterquartileRange |
| log-sigma-5-0-mm-3D_glrlm_RunVariance |
| log-sigma-5-0-mm-3D_glszm_LargeAreaEmphasis |
| log-sigma-5-0-mm-3D_glszm_LargeAreaHighGrayLevelEmphasis |
| log-sigma-5-0-mm-3D_glszm_ZoneEntropy |
| log-sigma-5-0-mm-3D_ngtdm_Busyness |
| wavelet-HLL_firstorder_Kurtosis |
| wavelet-LLL_glszm_LowGrayLevelZoneEmphasis |
| wavelet-LLL_glszm_SmallAreaEmphasis |
| \| wavelet-LLL_gldm_SmallDependenceLowGrayLevelEmphasis \| \| --- \| |

1. **T2WI radiomics signature**

The features of ICC calculated by segmentation difference were initially screened to obtain 1104 features with ICC ≥ 0.8. Then, PCC analysis was performed on the remaining radiomics features to obtain a feature set with relatively small redundancy (the correlation coefficient threshold was set to 0.99) and 667 features were obtained. After standardizing the obtained feature set by Minmax or Zscore algorithm, the RFE algorithm was used to find the optimal feature subset. The final model contained 6 features and the classifier was K-Nearest Neighbor (KNN).

| original_firstorder_Minimum |
| --- |
| original_glszm_SmallAreaEmphasis |
| log-sigma-3-0-mm-3D_glszm_SizeZoneNonUniformityNormalized |
| wavelet-LHL_glszm_SizeZoneNonUniformityNormalized |
| wavelet-LHL_gldm_DependenceNonUniformityNormalized |
| wavelet-LHH_glcm_ClusterShade |

**Table S3** Diagnostic performance of MRI-reported LNM

|  |  | Pathological diagnosis | |  | Diagnostic performance measures | | | | |
| --- | --- | --- | --- | --- | --- | --- | --- | --- | --- |
|  |  | pN- | pN+ |  | Sensitivity | Specificity | Accuracy | PPV | NPV |
| MRI-reported LNM | cN- | 91 | 29 |  | 70.4% | 66.9% | 68.4% | 60.5% | 75.8% |
|  | cN+ | 45 | 69 |  |  |  |  |  |  |

PPV: positive predictive value; NPV: negative predictive value; LNM, lymph node metastasis; pN-: patients with no lymph node metastases confirmed by pathology; pN+: patients with one or more lymph node metastases confirmed by pathology; cN-: patients with MRI reports indicating no lymph node metastasis.; cN+: patients with MRI reports indicating lymph node metastasis
